# 811. Is Obesity a Risk Factor for Skin Graft Loss in Burn Patients?

**DOI:** 10.1093/jbcr/irag033.250

**Published:** 2026-04-06

**Authors:** Charlotte Rogers, Nicholas Larson, Marah Kays, Rachael Rivard

**Affiliations:** Regions Hospital Burn Center, Saint Paul, MN; Regions Hospital Burn Center, Minneapolis, MN; Regions Hospital, Minneapolis, MN

## Abstract

**Introduction:**

At our ABA verified burn center, we noticed that obese patients may experience a higher rate of skin graft loss than non-obese patients. Recently, studies have demonstrated that in obesity, hypertrophy of adipose cells leads to poor tissue vascularity. In addition to other factors that make treatment of obese burn patients challenging, when skin grafting is needed for obese patients, a poorly vascularized adipose wound bed may lead to higher rates of skin graft loss.

**Methods:**

We designed a retrospective study to determine if obesity is a risk factor for skin graft loss, with secondary aims to evaluate the difference in outcomes between patients of varying BMI, including frequency of burn wound infection, hospital length of stay, number of surgeries required to achieve definitive wound closure. We analyzed all patients who underwent skin grafting for burn treatment at our ABA verified burn center from 2021-2023. Charts were queried for "graft loss." Multivariable logistic regression was used to estimate the adjusted odds of graft loss associated with increasing BMI. To identify BMI thresholds across burn sizes where refined treatment may be necessary to reduce graft loss, the sensitivity and specificity of BMI for predicting graft loss was determined for small (0-9.9% TBSA), medium (10-19.9% TBSA), and large (≥20% TBSA) burns.

**Results:**

Retrospective chart review identified 202 patients meeting inclusion criteria; 110 patients experienced some degree of graft loss, while 92 patients did not have documented graft loss. Multivariate model found that increased BMI is significantly associated with graft loss. As BMI increases, the likelihood of graft loss increases in a linear fashion. Across all burn sizes, a BMI of 28.5 was identified as the intersection between sensitivity/specificity and Youden peak. While increased BMI was associated with graft loss, it's impacts on frequency of burn wound infection, hospital length of stay, and number of surgeries to achieve wound closure were not significant. While increasing BMI was associated with graft loss, the greatest predictor of graft loss was burn size ≥20% TBSA. Compared to patients with smaller burns (0-9.9% TBSA), the adjusted odds of graft loss in large burns (≥20% TBSA) was 3.59 times greater (95%CI 1.44-8.91).

**Conclusions:**

Increasing BMI was found to be a significant predictor of skin graft loss in burn patients.

**Applicability of Research to Practice:**

Due to an increased risk of graft loss, we might consider alternative treatment strategies than primary skin grafting for patients with a BMI above 28.5. These strategies might include a staged surgical approach to first optimize wound bed perfusion, with either wound vac therapy, skin substitute, or fascial excision prior to skin grafting.

**Funding for the study:**

N/A.

